# Supplementary material for: Complexome profiling of the Chlamydomonas psb28 mutant reveals TEF5 as an early PSII assembly factor
Source: Plant Cell. 2025 Mar 17;37(6):koaf055. doi: 10.1093/plcell/koaf055 (PMC12164590; doi:10.1093/plcell/koaf055)
Supplement: koaf055_Supplementary_Data [file koaf055_supplementary_data.zip › psb28_interactive.zip!/plots/intensity/qualityPlots/peptides/EFT2_Cre10.g435500.t1.1.html]

### Cre10.g435500.t1.1

Trivial name: EFT2  
SeqCoverage [median]: 8.90  
Mass: 40.63 kDa  
  
 

| Sequence | MissedCleavage | Mass [Da] | wtIntensity | muIntensity | Score |
| --- | --- | --- | --- | --- | --- |
| AGGGDFAAEVAR | 0 | 1119.53 | 370.63 | 349.70 | 83.86 |
| LASVVVLAPEPDDKAQPLDAAGR | 1 | 2331.24 | 0.00 | 574.64 | 60.56 |
|  |  |  | 370.6 | 924.3 |  |
